# General movement assessments in neonates born with congenital gastrointestinal anomalies: a single site, retrospective study

**DOI:** 10.1038/s41372-024-02207-8

**Published:** 2025-02-21

**Authors:** Lisa Soumekh, Samantha Bell, Sandra L. Espinosa, Tristan Grogan, Kalpashri Kesavan, Kara L. Calkins

**Affiliations:** 1 Department of Pediatrics, David Geffen School of Medicine, University of California Los Angeles, Los Angeles, CA USA; 2 Department of Medicine Statistics Core, David Geffen School of Medicine, University of California Los Angeles, Los Angeles, CA USA; 3 Department of Pediatrics, Division of Neonatology, University of California Los Angeles, Los Angeles, CA USA

**Keywords:** Risk factors, Medical research

## Abstract

**Objective:**

We aimed to characterize general movements in neonates with congenital gastrointestinal anomalies and to compare them to general movements in extremely low birth weight (ELBW) infants.

**Study design:**

This was a retrospective, single-site study. Subjects were divided into two groups: those with gastrointestinal (GI) anomalies and ELBW infants (birth weight <1 kg). The primary outcome was general movement assessments.

**Result:**

Ninety-six infants were included (*n* = 51, GI group and *n* = 45, ELBW group). The rates of abnormal general movements between the groups were comparable (writhing movements: 61% vs. 59%, *p* = 0.89; fidgety movements: 20% vs. 36%, *p* = 0.13). Writhing movements were different (100% poor repertoire, 0% cramped synchronous in the GI group vs. 50% poor repertoire and 50% cramped synchronous in the ELBW group, *p* < 0.001).

**Conclusion:**

Infants with gastrointestinal anomalies are at risk for abnormal general movements. Abnormal fidgety general movements may be an early biomarker for future motor deficits.

## Introduction

Neonates born with congenital gastrointestinal anomalies are at risk for neurodevelopmental impairment. They are often born premature, require prolonged hospital stays, multiple surgeries, anesthesia, and mechanical ventilation, and are at high risk for poor growth, malnutrition, and infections—all of which adversely impact neurodevelopment [1–5].

In comparison to premature infants, there is a paucity of literature exploring the neurodevelopment of infants born with gastrointestinal anomalies [4, 6, 7]. Studies have demonstrated that extremely low birth weight infants (ELBW, birth weight < 1 kg) are at high risk for motor, cognitive, and behavioral impairments [8–11]. Most ELBW infants are followed after hospital discharge from the neonatal intensive care unit (NICU) with serial neurodevelopmental assessments [12]. However, follow-up for infants with gastrointestinal anomalies is not standard practice. Research has demonstrated that early identification and intervention decreases the risk of developmental disability [13–16].

Identifying infants at risk of neurodevelopmental impairment requires practical, reliable, and cost-effective assessments. Prechtl’s General Movements is a low-cost, high-yield tool used to predict motor delays. It is utilized to predict cerebral palsy as early as three to four months post-term [17–20]. General movements are motor patterns endogenously produced by the young, adapting nervous system. The infant is observed in a supine position while awake at two stages of development [17]. When the nervous system is impaired, general movement quality changes. Writhing movements are observed at both preterm and term age and are categorized as either normal writhing, poor repertoire, or cramped synchronous. Fidgety movements are observed around three to four months post-term age. They are categorized as normal, abnormal, or absent.

General movement assessments are highly sensitive and specific in predicting cerebral palsy with studies reporting a sensitivity as high as 98–100% [18, 21]. General movement assessments, particularly at the fidgety age, have been found to have a higher sensitivity and specificity than common imaging modalities including magnetic resonance imaging and head ultrasounds [18, 22]. In comparison to magnetic resonance imaging and head ultrasound, general movements are easier to collect and more cost-effective. Caregivers can videotape the infant at home and send these videos to certified practitioners for review.

Since there is a lack of literature on the neurodevelopmental outcomes in infants born with congenital gastrointestinal anomalies, our study aimed to characterize general movements in this population and compare them to another high-risk population that is cared for in the NICU, ELBW infants. With a better understanding, our ultimate goal is to determine if general movement assessments have a role in predicting long-term neurodevelopmental outcomes in this population.

## Materials and methods

This is a retrospective, single-site study that included infants born at the University of California Los Angeles between March 2018 and December 2022 and admitted to the NICU. Inclusion criteria included those born with a congenital gastrointestinal anomaly or birth weight < 1 kg (ELBW). Congenital gastrointestinal anomalies included anatomical anomalies that were present at birth including tracheoesophageal fistula, abdominal wall defects, congenital diaphragmatic hernia, intestinal atresia, Hirschsprung’s disease, and anorectal malformations. Patients that were included underwent at least one general movement assessment. Patients who expired before hospital discharge or those with a known chromosomal anomaly were excluded from the study. Included patients were divided into two groups, those with a congenital gastrointestinal anomaly (GI group) and those who were ELBW (ELBW group). This study was approved by our local Institutional Review Board.

Demographic data was collected from infant and maternal electronic medical records. Data collected from the subject’s hospital courses included variables that are known to impact neurodevelopment such as fetal growth restriction, early and late onset sepsis, duration of parental nutrition, mechanical ventilation, and intraventricular hemorrhage.

Starting in 2018, general movement assessments were obtained for infants admitted to the NICU that did not qualify for long-term neurodevelopmental follow-up. In 2020, the program was adjusted to screen all infants that required seven or more days in the NICU. General movement assessments were administered at two ages. Writhing general movements were observed at 36–46 weeks corrected gestational age while patients were in the NICU. Fidgety general movements were observed at 3–4 months post-term age as part of a multidisciplinary clinic appointment or in the inpatient setting if the infant was hospitalized. For both assessments, infants were recorded by video. Videos were reviewed by health care professionals certified in general movement assessments. Writhing movements were categorized as normal or abnormal. Abnormal writhing general movements were further categorized as poor repertoire or cramped synchronous. Fidgety general movements were characterized as normal fidgety, absent fidgety, or abnormal fidgety.

### Statistics

Patient characteristics and study variables were summarized by group (GI group and ELBW group) using mean (SD) or frequency (%) as appropriate. These variables were compared using the t-test or chi-square test, as appropriate. To assess the association between abnormal general movement assessment (abnormal writhing/abnormal fidgety) and gestational age and birth weight, univariable logistic regression models were utilized. Statistical analyses were conducted using R V4.1.0 (www.r-project.org; Vienna, AU), and *p*-values < 0.05 were considered statistically significant.

## Results

Ninety-six infants met inclusion criteria. There were 51 subjects in the GI group and 45 subjects in the ELBW group. The GI group included a heterogenous group of congenital anomalies: tracheoesophageal fistula (22%, *n* = 11), gastroschisis (22%, *n* = 11), congenital diaphragmatic hernia (16%, *n* = 8), anorectal malformations (12%, *n* = 6), Hirschsprung’s disease (6%, *n* = 3), and other abnormalities (22%, *n* = 12).

When the ELBW group was compared to the GI group, the mean (SD) gestational age and birth weight were significantly different (26 (2) weeks vs. 35 (4.7) weeks, *p* < 0.001 and 715 (160.5) g vs. 2408 (1001.2) g, *p* < 0.001). The ELBW group also had higher rates of co-morbidities associated with prematurity, including necrotizing enterocolitis (13% vs. 2%, *p* = 0.048), late onset sepsis (24% vs. 4%, *p* = 0.003), and all grades of intraventricular hemorrhages (47% vs. 10%, *p* < 0.001). The GI group underwent more surgeries (2 (1) vs 1 (1), *p* < 0.001), including abdominal surgeries (1 (0.7) vs 0.4 (0.7), *p* < 0.001) (Table 1).

**Table 1:** Maternal and neonatal characteristics of extremely low birthweight infants and those born with congenital gastrointestinal anomalies.

	GI (*n* = 51)	ELBW (*n* = 45)	*p* value
Gestational age (weeks)	35 (4.7)	26 (2.0)	<0.001
Female	41% (21)	56% (25)	0.16
Birth weight (g)	2408 (1001.2)	715 (160.5)	<0.001
Birth length (cm)	44 (7.4)	32 (2.4)	<0.001
Birth head circumference (cm)	31 (4.8)	23 (1.8)	<0.001
Birth weight z-score	−0.5 (1.1)	−0.6 (1.3)	0.74
Birth length z-score	−0.3 (1.1)	−0.6 (1.4)	0.25
Cesarean section	49% (25)	84% (38)	<0.001
Maternal age (years)	30 (7.0)	35 (6.5)	0.003
Maternal gravida	2 (1.7)	3 (2.1)	0.46
Maternal parity	2 (1.1)	2 (1.5)	0.68
Maternal drug use	20% (10)	2% (1)	0.03
Maternal tobacco use	14% (7)	7% (3)	0.42
Maternal alcohol use	8% (4)	11% (5)	0.90
Intraamniotic infection	10% (5)	4% (2)	0.44
Small for gestational age	26% (13)	27% (12)	0.90
Fetal growth restriction	32% (13)	41% (17)	0.41
Length of stay (days)	52 (46.1)	104 (26.4)	<0.001
Intubation (days)	19 (20.5)	35 (21.7)	0.003
Parenteral nutrition (days)	28 (30.1)	25 (20.6)	0.49
Central line (days)	30 (31.1)	35 (37.5)	0.49
NEC (Stage II or III)^a^	2% (1)	13% (6)	0.05
Early onset sepsis	0	2% (1)	0.47
Late onset sepsis	4% (2)	24% (11)	0.003
Meningitis	0	2% (1)	0.47
IVH (any grade)	10% (5)	47% (21)	<0.001
Surgeries	2 (1)	1 (1)	<0.001
Abdominal surgeries	1 (0.7)	0.4 (0.7)	<0.001

Data presented as mean (SD) or *n* (%). Intraventricular hemorrhage (IVH) includes grades I-IV. Small for gestational age is a birth weight of less than 10th percentile at birth. Fetal growth restriction is an estimated fetal weight below 10th percentile on prenatal ultrasound. Necrotizing enterocolitis (NEC) is defined as Bell’s staging two or greater [1]. Early onset sepsis is a positive blood culture before 72 h of age. Late onset sepsis is a positive blood culture after 72 h of age.

*GI* gastrointestinal, *ELBW* extremely low birth weight.

^a^Reed and Dimmitt [38].

In the GI group, 66% (*n* = 34) of infants had a writhing general movement assessment, 64% (*n* = 33) had a fidgety general movement assessment, and 23% (*n* = 12) underwent both assessments. In the ELBW group, 82% (*n* = 37) had a writhing general movement assessment, 77% (*n* = 35) had a fidgety general movement assessment, and 62% (*n* = 28) underwent both assessments. Neonates in the GI group demonstrated abnormal general movements. There was no significant difference in the rate of abnormal general movements between the two groups. For the writhing movements, 58% and 59% of assessments were abnormal for the GI group and ELBW group, respectively (*p* = 0.96). For the fidgety movements, 15% of the assessments were abnormal for the GI group vs. 34% for the ELBW group were abnormal (*p* = 0.07).

**Fig. 1:**
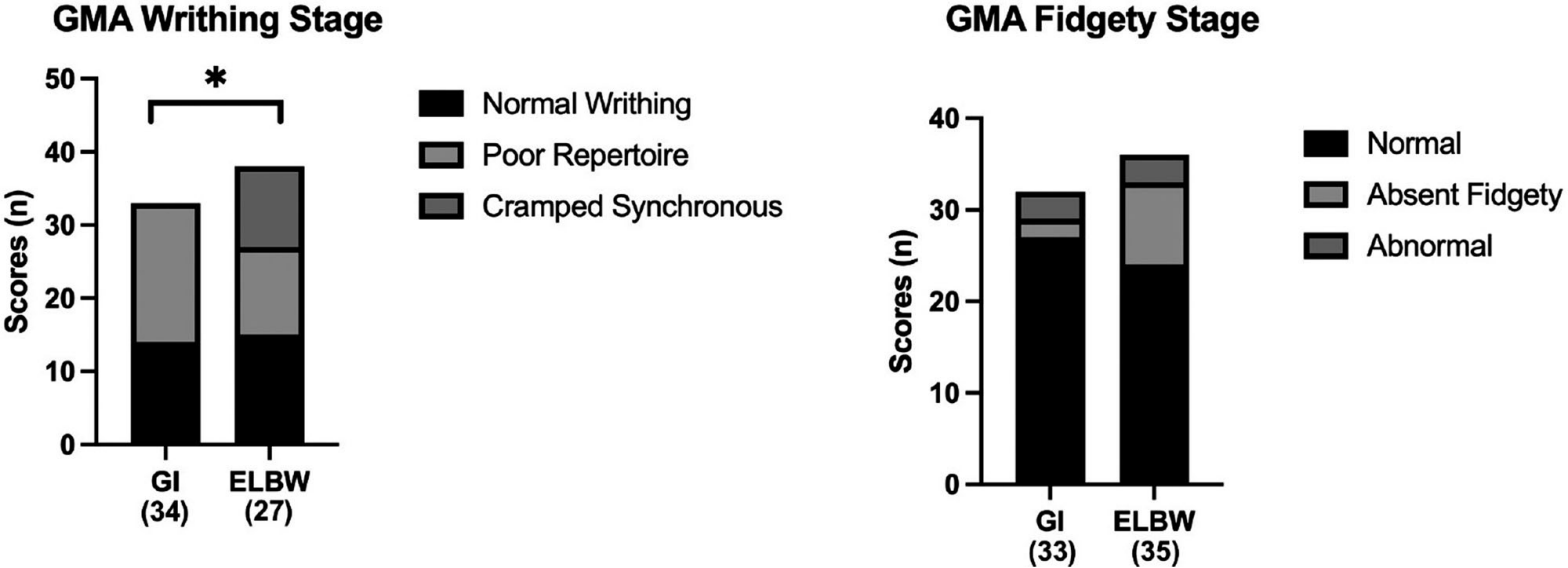
General movement assessments. GMA general movement assessments, GI gastrointestinal, ELBW extremely low birthweight.

Writhing general movements were significantly different between the two groups (*p* < 0.001). Forty-one percent (*n* = 14) of the GI group had a normal writhing general movement and 59% (*n* = 20) of the general movements were characterized as poor repertoire. The ELBW group demonstrated a different pattern; 40% (*n* = 15) had a normal writhing general movement; 30% (*n* = 11) of the general movement were characterized as poor repertoire; and 30% (*n* = 11) were characterized as cramped synchronous. Fidgety general movements were not significantly different between the two groups (*p* = 0.076) (Fig. 1).

Within each group, we compared the characteristics of neonates with abnormal versus normal general movements for each developmental stage. For writhing movements, infants with congenital gastrointestinal anomalies who had abnormal general movements were intubated longer than those with normal general movements (30 (27) days vs. 10 (11) days, *p* = 0.045). In the ELBW group, infants with abnormal general movements were born at a younger gestational age (25 (2) weeks vs. 27 (2) weeks, *p* = 0.021), were intubated longer (45 (18) days vs. 22 (18) days, *p* < 0.001), and had higher rates of all grades of intraventricular hemorrhage (64% vs. 27%, *p* = 0.027). In both groups, there was no significant difference in the number of surgeries between infants with abnormal and normal general movements (ELBW group: 0.63 (0.95) vs. 0.53 (0.74), *p* = 0.72, GI group 1.8 (0.95) vs 1.4 (0.84), *p* = 0.18).

For fidgety movements within the GI group, those with abnormal or absent fidgety movements were born at a younger gestational age (30 (5.3) weeks vs. 36 (4.7) weeks, *p* = 0.026), lower birth weight (1263 (811) g vs. 2538 (989) g, *p* = 0.011), and had higher rates of fetal growth restriction (80% vs. 23% *p* = 0.03) compared to those with normal fidgety movements. In the ELBW group, those with abnormal or absent fidgety movements had lower birth weights (646 (183) g vs. 766 (137) g, *p* = 0.035), were intubated longer (45 (23) days vs. 27(23) days, *p* = 0.039), and had higher rates of all grades of intraventricular hemorrhage (67% vs 30%, *p* = 0.040) compared to those with normal fidgety movements. Again, in both groups, there was no significant difference in the number of surgeries between infants with abnormal and normal general movements (ELBW group 0.92 (1.2) vs 0.65 (0.83), *p* = 0.45, GI group 2 (1.2) vs 1.8 (1), *p* = 0.39).

Logistic regression analyses revealed increased odds of abnormal general movements with lower birth weights and younger gestational ages. In the GI group, the odds of abnormal or absent fidgety movements increased by 17% (OR 0.83, 95% CI 0.70–0.99, *p* = 0.036) for each week decrease in gestational age. The odds of abnormal or absent fidgety movements also increased by 50% (OR 0.50, 95% CI 0.28–0.88, *p* = 0.017) for each 500 g decrease in birth weight. In the ELBW group, the odds of abnormal writhing movements increased by 37% (OR 0.63, 95% CI 0.41–0.97, *p* = 0.036) for each week decrease in gestational age. The odds of abnormal or absent fidgety movements increased by 93% (OR 0.07, 95% CI 0.01–0.88, *p* = 0.40) for every 500 g decrease in birth weight (Table 2).

**Table 2:** Association between birth weight and gestational age with abnormal writhing movements (abnormal writhing) and abnormal fidgety movements (abnormal or absent fidgety).

		Abnormal writhing		Abnormal or absent fidgety	
		OR (95% CI)	*p* value	OR (95% CI)	*p* value
GI	Birth Weight (0.5 kg)	1.06 (0.77–1.46)	0.74	0.50 (0.28–0.88)	0.02
	Gestational Age (week)	0.99 (0.87–1.14)	0.93	0.83 (0.70–0.99)	0.04
ELBW	Birth Weight (0.5 kg)	0.34 (0.04–2.92)	0.32	0.07 (0.01–0.88)	0.04
	Gestational Age (week)	0.63 (0.41–0.97)	0.04	0.72 (0.48–1.06)	0.09

*GI* gastrointestinal, *ELBW* extremely low birth weight.

## Discussion

In this study, infants born with gastrointestinal anomalies had the same rate of abnormal general movements as ELBW infants. The two groups demonstrated a significantly different pattern for writhing movements. As anticipated, in both groups, abnormal general movements were associated with prematurity and low birth weights. Given these findings, further research is needed to determine if general movements in the neonatal gastrointestinal population correlate with long-term neurodevelopmental outcomes.

Studies have examined the neurodevelopmental outcomes of those born with congenital gastrointestinal anomalies and have found that these infants are at risk for long-term neurological dysfunction [2, 4, 5, 7]. Roorda et al. performed a systemic review and meta-analysis of studies examining cognitive, motor, and language outcomes of those born with congenital gastrointestinal anomalies. Overall, these infants were found to have significant cognitive, motor, and language impairments [23]. In a prospective cohort study of infants born with congenital anomalies who underwent cardiac and noncardiac surgery at 1 and 3 years of age, the study population scored lower than the general population on the Bayley Scales Infant and Toddler Development-III assessment [4, 5]. Studies have also examined specific congenital anomalies. In one study, infants with tracheoesophageal fistulas had lower intelligence quotients and were more likely to have emotional and behavioral problems compared to controls [7]. Friedman et al. found that those with congenital diaphragmatic hernias had higher rates of motor and language delays up to 3 years old [24]. Considering the literature, long-term neurodevelopmental follow-up for infants born with congenital gastrointestinal anomalies appears to be warranted.

Historically, general movements have been most reliable in predicting motor outcomes in very preterm infants [25]. Cramped synchronous movements at the writhing stage followed by absent fidgety movements are the most sensitive for predicting cerebral palsy [19, 20]. Studies investigating the utility of general movements for predicting mild neurological deficits have had variable results. This is likely because of heterogenous study populations, different neurodevelopmental assessments, and the availability of general movement assessments. Darsaklis et al. undertook a systemic review exploring the predictive value of general movements for neurodevelopmental outcomes at multiple ages. They found that the sensitivities and specificities for mild neurological deficits were lower than the sensitivities and specificities for cerebral palsy [21]. For writhing movements at 12–23 months, the sensitivities ranged from 75 to 100% for predicting cerebral palsy. Likewise, the sensitivity for writhing movements was 75–86% in predicting scores on standardized developmental assessments. Fidgety movements had a high sensitivity for cerebral palsy (100%), but lower sensitivity (38–62.5%) in predicting scores on standardized developmental assessments [21].

Further studies will be needed to determine the significance of abnormal general movements within the gastrointestinal population. In our study, the gastrointestinal group had significantly higher rates of poor repertoire writhing movements compared to ELBW infants. The long-term significance of this finding remains unclear. Many infants with poor repertoire writhing movements will have normal fidgety movements and normal development [17]. In comparison, infants with cramped synchronous writhing movements have higher rates of abnormal or absent fidgety movements and developmental impairments [17, 26, 27]. In a study examining general movements in the neonatal surgical population, 84% of infants with abnormal writhing movements went on to have normal fidgety movements. While those with normal fidgety movements did not demonstrate cerebral palsy at 1 year of age, 48% had developmental delays [26]. These transitory changes in writhing general movements may reflect short-term exogenous factors such as infections, postnatal steroid therapy, analgesic medications, and the presence of a patent ductus arteriosus rather than a brain injury that alters long-term development [2, 28, 29].

In our study, infants with abnormal general movements were born at younger gestational ages, lower birth weights and were intubated for longer periods of time. ELBW infants with abnormal general movements also had higher rates of all grades of intraventricular hemorrhage. These findings are consistent with the literature [2,28–33]. Maeda et al. explored the association of general movement quality and brain morphological development using magnetic resonance imaging. Mechanical ventilation was correlated with abnormal general movements [32]. In another study, investigators noted the absence of intraventricular hemorrhage grades I-II reduced the chances of an abnormal general movement by 77%. Each day of invasive mechanical ventilation increased the odds of an abnormal general movement by 1.11 (95% CI 1.01–1.22, *p* = 0.025) [31]. While intraventricular hemorrhage causes direct injury to the brain, mechanical ventilation affects brain oxygenation and metabolism.

Twenty-five percent of infants born with gastrointestinal anomalies in our study were fetal growth restricted. These infants had significantly higher rates of abnormal or absent fidgety general movements. Fetal growth restriction has been shown to directly affect brain development through multiple mechanisms [34]. Fetal growth restriction is a reflection of placental insufficiency resulting in chronic fetal hypoxia. Fetal growth restriction is associated with reduced total brain volume, altered brain structures, and a decreased total number of cells [34].

Multiple factors likely contribute to the development of abnormal movements in those born with congenital gastrointestinal anomalies. These infants are exposed to various perinatal risk factors including environmental hazards (i.e., maternal smoking) and are at increased risk for non-gastrointestinal congenital anomalies [35, 36]. Central nervous system malformations directly affect brain architecture and can alter function. None of the subjects included in our study had known central nervous system abnormalities. This finding is limited. Most infants underwent cranial ultrasounds; however only one-third underwent a brain MRI. It would be interesting for future studies to better characterize the frequency of cranial abnormalities in this population.

High rates of surgical intervention in those born with congenital gastrointestinal anomalies also likely contributes to abnormal general movements. The GI group in our study underwent significantly more surgeries than the ELBW group. Infants with abnormal general movements did not have higher rates of surgery when compared to those with normal general movements. Surgery carries multiple risks including anesthesia, intubation, prolonged immobilization, infections, and surgical complications. Prior studies have found a correlation between surgeries and developmental outcomes. In a systemic review and metanalysis of neurodevelopmental outcomes of patients with congenital gastrointestinal malformations, there was a higher rate of neurodevelopment impairment compared with normative data or healthy controls (d = −0.49, 95% CI −0.61 to −0.38, *p* < 0.001). Poor outcomes were related to the number of surgeries and length of hospital stay (b = −0.005, *p* < 0.001 and b = −0.1371, *p* = 0.003) [23]. Immobilization following surgery delays exposure to core abdominal strengthening exercises and environmental exploration that promotes development.

Furthermore, animal studies have demonstrated that anesthetic agents have a direct neurotoxic effect on neurons. Rats exposed to drugs that block NMDA glutaminergic receptors or drugs that potentiate GABA receptors have widespread apoptotic neurodegeneration [37]. In children, cumulative doses of anesthetics increases the risk of learning disorders (hazard ratio for 2 anesthetics = 1.59, 95% CI 1.06–2.37, and hazard ratio for ≥3 anesthetics = 2.60, 95% CI 1.60–4.24) [3].

We acknowledge that this study has multiple limitations. This is a single-site, retrospective study with a small sample size. Gastrointestinal congenital anomalies are also heterogenous. With a larger sample size, specific sub-analyses would have been possible. We recognize that there are multiple differences between our population of interest, infants with gastrointestinal disorders, and our comparison group, ELBW infants. Our goal was to compare infants with gastrointestinal anomalies to a population with a known risk for abnormal general movements and neurodevelopmental impairment. Not all subjects had imaging data. We were unable to accurately quantify the type and duration of anesthetic exposures due to the retrospective nature of this study. Lastly, we lack long-term neurodevelopmental data and general movement assessments are not performed consistently on all subjects. Missing data may have altered our results.

In conclusion, in our study, 58% of the infants with congenital gastrointestinal anomalies had abnormal writhing movements and 15% had abnormal fidgety movements. We speculate that infants born with congenital gastrointestinal anomalies are at high risk for future neurodevelopmental impairments due to prematurity, low birth weight, and need for prolonged intubation and surgeries. Identifying those at the highest risk is vital to ensuring long-term follow-up. With improved long-term follow-up, practitioners have the opportunity to reduce poor neurodevelopmental outcomes through earlier interventions. Abnormal general movement may be an early biomarker for future deficits for infants born with congenital gastrointestinal anomalies.

## Data Availability

The datasets analyzed during the current study are available from the corresponding author on request.
